# Are happier adolescents more willing to protect the environment? Empirical evidence from Programme for International Student Assessment 2018

**DOI:** 10.3389/fpsyg.2023.1157409

**Published:** 2023-04-12

**Authors:** Min Zhang, Weidong Zhang, Yong Shi

**Affiliations:** ^1^The Co-Innovation Center for Social Governance of Urban and Rural Communities in Hubei Province, Zhongnan University of Economics and Law, Wuhan, Hubei, China; ^2^School of Business Administration, Zhongnan University of Economics and Law, Wuhan, Hubei, China; ^3^School of Accounting, Zhongnan University of Economics and Law, Wuhan, Hubei, China

**Keywords:** subjective wellbeing, pro-environmental behaviors (PEBs), self-efficacy, peer relationships, multicultural values, adolescents

## Abstract

A large number of existing studies have discussed the potential factors affecting pro-environmental behaviors (PEBs) in adolescents. However, few studies have focused on the possible impact of adolescents’ subjective wellbeing (SWB) on their PEBs. Why and how adolescents’ SWB affects their PEBs remains a puzzle. To unravel this puzzle, this paper aims to establish a suitable instrumental variable (IV) to correctly estimate the contribution of adolescents’ SWB to their PEBs. Using the international data from the Organisation for Economic Co-operation and Development (OECD) ‘s Programme for International Student Assessment 2018, we construct a unique dataset of eight countries or economies, which includes 56,374 samples related to the SWB and PEBs of 15-year-old students. In this paper, the days of physical education classes in school per week are used as the IV. Through a two-stage least squares method, we find that the contribution of adolescents’ SWB to PEBs is significantly positive. We also find that the pathway by which SWB improves PEBs works through adolescents’ self-efficacy. Furthermore, the results indicate that the positive impact of SWB on PEBs is more pronounced among adolescents with better peer relationships and stronger multicultural values. Our findings highlight the influence of positive affects in cultivating adolescent’ PEBs and the importance of growing up surrounded by happiness.

## Introduction

Environmental protection and adolescents’ healthy growth are significant issues of global sustainable development (WHO, 2015). Adolescents face the enormous challenge of environmental degradation and are the future decision-makers and actors in environmental protection (Balunde et al., 2020; Poškus, 2020). Implementing behaviors that benefit the natural environment or reduce damage to the natural environment are considered pro-environmental behaviors (PEBs) (Kollmuss and Agyeman, 2002; Lange and Dewitte, 2019). However, a large number of studies have shown that adolescents are not as interested in environmental protection (Negev et al., 2008; Uitto and Saloranta, 2010; Uitto et al., 2011; Wray-Lake et al., 2016, 2017). Environmentally friendly attitudes decline as children enter adolescents. Thus, the questions about the influencing factors and mechanism of adolescents’ PEBs are of primary importance. Nonetheless, our understanding of PEBs in adolescents is still limited (Balunde et al., 2020).

Actually, some scholars have found that adolescents have a weaker preference for the natural environment. In particular, they are significantly less interested in and concerned about environmental issues than children and adults. From ages 10 to 19, adolescence is a unique stage of human development between childhood and adulthood (WHO, 2022). Adolescence has been described as a period of “moratorium,” a period of freedom from responsibility (Erikson, 1994). Moreover, if adolescents are left out of responsibility for too long, they may not learn to manage their own lives or take on social responsibility. Kahn and Kellert (2002) describe adolescence, particularly the 14−16 age group, as a “time out” in preference for the natural environment. Olsson and Gericke, 2016 refer to this period as the “adolescent dip.”

Adolescent PEBs, defined as the responsible behavior of students toward environmental sustainability, are of growing interest to policymakers and educators worldwide (UNECE, 2005; Association for Physical Education, 2015; WHO, 2015; Group of Experts for the Pact, 2017; OECD, 2019). It is significant to explore the predictor of adolescents’ PEBs as today’s adolescents are tomorrow’s workforce (Group of Experts for the Pact, 2017; OECD, 2019). The Theory of Planned Behavior (Ajzen, 1991), the Norm-Activation-Theory (Schwartz and Howard, 1981), and the Value-Belief-Norm-Theory (Stern, 2000), the most commonly used theories in the field of environmental psychology, provide great insight into the prediction of intention on environmental behavior (Klöckner, 2013). However, it has been argued that these models ignore the potential influence of emotions on behaviors (Kim et al., 2013; Han et al., 2017; Tian and Liu, 2022). Recently, an emerging school of thought has explored the impact of subjective wellbeing (SWB) on PEBs from a positive psychology perspective (Sulemana, 2016; Wang and Kang, 2019; Kushlev et al., 2021; Nguyen et al., 2022; Ouyang et al., 2022). Specifically, there is a significant correlation between people’s SWBs and their PEBs. Yet, this relationship has only been tested in adults and has not been tested in adolescents. Unlike adults, adolescents’ values, behaviors, and ethics are still developing and unstable (Balunde et al., 2020). Can then, adolescents’ SWB promote their PEBs?

Given the significant differences in the performance of adolescents and adults in PEBs, there is a strong need to examine the impact of adolescents’ SWB on their PEBs. In response to a growing call for research to determine whether increases in SWB enhance the likelihood that adolescents will engage in PEBs, there are still three gaps in the existing literature that need to be addressed.

Firstly, our understanding of the relationship between SWB and PEBs is still limited. On the one hand, some scholars suggest that people are happier and more willing to support policies that promote environmental sustainability (Gowdy, 2005; Duroy, 2008; Kushlev et al., 2021). People with a higher SWB are more willing to sacrifice their interests to protect the environment or are more willing to change their lifestyle to reduce environmental damage (Sulemana, 2016; Solano-Pinto et al., 2020; Welsch, 2020; Kushlev et al., 2021). However, other scholars believe that too much SWB may interfere with motivations (Diener et al., 2018). It has also been referring to the Pollyanna hypothesis, that people with higher SWB are usually more satisfied with their lives and lose the incentive to make the world a better place (Kushlev et al., 2020).

Secondly, the direction of causality between SWBs and PEBs is widely debated. More specifically, whether PEBs make people happier or happier people are more likely to protect the environment, and there are different arguments regarding this question (see Table 1). Much of the early research looked at SWBs as outcomes, and the literature suggested that PEBs could give people a sense of achievement and thus enhance their SWB. Nevertheless, a growing body of recent literature considered SWB as a predictor of PEBs. This plausible reverse causality suggests that endogeneity problems may exist. Therefore, the estimates obtained by the ordinary least squares method may not be reliable.

**TABLE 1:** Is SWB the outcome or the predictor of PEBs?

The role of SWB	References
SWB as the outcome	Ferreira and Moro (2010), Gu et al. (2015), Kaida and Kaida (2016b,2019), Su et al. (2018), Zhang and Tu (2021) etc.
SWB as the predictor	Sulemana (2016), Wang and Kang (2019)Kushlev et al. (2020), Kushlev et al. (2021), Nguyen et al. (2022), Ouyang et al. (2022) etc.

To accurately estimate the effect of SWB on PEBs, a suitable instrumental variable (IV) must be found. The existing literature can be regarded as a process of a constant search for IV for SWB. Sulemana (2016) used “tolerance and respect for other people,” while Wang and Kang (2019) selected weather change as an IV and found that residents, where there were unexpected parts of sunshine (UPS), were more likely to report higher levels of life satisfaction. Ouyang et al. (2022) suggested choosing the frequency of individuals’ recreational activities, such as listening to music in their spare time, as an IV. Nevertheless, these IVs did not address the endogeneity of SWB very well. Sulemana (2016) and Ouyang et al. (2022) chose IVs that were still subjective and not exogenous. Wang and Kang (2019) used the exogenous variable of weather, but the weather also objectively influenced people’s accessibility to participation in environmental activities.

Last but not least, most studies use single-item measures of SWB and lack multi-item measures. For instance, in the literature examining the effect of SWB on PEBs, Sulemana (2016), Wang and Kang (2019), Ouyang et al. (2022) all explored one question item to measure SWB. While this approach is easy to administer, it is conceptually unsatisfactory, potentially invalid, and unreliable, especially in cross-country comparisons (OECD, 2019).

To shed light on these puzzles, we employ the OECD’s (2018) Programme for International Student Assessment (PISA 2018) data, which measured 15-year-olds’ wellbeing and ability to use knowledge and skills to meet real-life challenges. In the data of PISA 2018, adolescents’ SWBs and their PEBs were measured using a multi-item questionnaire administered in eight countries or economies. Firstly, the ordered probit model is used to estimate the effect of adolescents’ SWB on their PEBs. Additionally, we implement a two-stage least squares (2SLS) estimator by using the days of physical education (PE) classes per week at school as an IV for adolescents’ SWB. Lastly, we also explore the potential mechanisms through which adolescents’ SWB might affect their PEBs.

Drawing on the international dataset of PISA 2018, we find that SWB in adolescents has a positive effect on PEBs. That is to say, for adolescents, the happier they are, the more willing they are to exhibit PEBs. Existing literature has shown that there is a bidirectional causal relationship between an individual’s PEBs and their SWB (Sulemana, 2016; Diener et al., 2018; Wang and Kang, 2019; Ouyang et al., 2022). Thus, we introduce the days of PE classes per week at school as an IV to effectively estimate the contribution of adolescents’ SWB to their PEBs, and the results remain robust. Additionally, we explore the mediating effects of self-efficacy on the relationship between SWB and PEBs. We use adolescents’ self-reported confidence in overcoming difficulties as a proxy for self-efficacy. The empirical results suggest self-efficacy accounts for some of the relationship between SWB and PEBs. Finally, we further investigate the heterogeneity of the different subgroups. We find that the positive effect of SWB on PEBs is more significant for adolescents who have more opportunities to get along with classmates in their leisure time after school. Moreover, multicultural values moderate the relationship between adolescents’ SWB and their PEBs. Specifically, the positive influence coefficient of SWB on PEBs is greater for adolescents with stronger multicultural values.

Our research makes several contributions. Firstly, we execute a methodology that address the endogeneity of adolescents’ SWB and estimate the contribution of adolescents’ SWB to their PEBs. This builds on the previous literature testing the correlation of SWB and PEBs in adults (Sulemana, 2016; Wang and Kang, 2019; Ouyang et al., 2022). We use a cross-national dataset to confirm that this relationship still holds in the adolescent cohort. Simultaneously, our findings provide a new perspective for addressing the issue of “adolescent dip” or “time out” (Otto and Kaiser, 2014; Olsson and Gericke, 2016). These findings provide significant references that governments can use to formulate policies on adolescent mental health and environmental education. Secondly, we provide new evidence for the theoretical exploration of PEBs from the perspective of positive psychology. As the positive emotion, SWB is a great predicator for PEBs. Adolescents’ SWB can improve PEBs through their increased sense of self-efficacy, providing new evidence for the role of positive emotions in Environmental psychology (Kim et al., 2013; Klöckner, 2013; Han et al., 2017; Tian and Liu, 2022). Thirdly, our research further reveals the mechanism of SWB’s influence on PEBs. Specifically, we find that adolescents’ peer relationships and multicultural values moderate the effect of SWB on PEBs. These findings help to provide insight into the specific ways and means by which SWBs affect PEBs.

The rest of the paper proceeds as follows. At first, we review the literature, outline our theoretical arguments, and lay out the empirical hypotheses. Next, we describe the data and variables, and specify our research design. Then, we present the empirical results from the benchmark regression, the robustness checks, the analysis of mediating and moderating effects. Lastly, we draw the discussions and the conclusions.

## Literature review

### SWB and PEBs of adolescents

Subjective wellbeing (SWB) refers to how people think and experience life in positive and negative ways (Diener et al., 2018). It is a cognitive evaluation of both people themselves and their own lives, which includes two basic components: life satisfaction and emotional experience (Diener, 1984). The former is an individual’s overall cognitive evaluation of their quality of life, that is, the degree to which they make a satisfactory judgment about their overall life. The latter refers to an individual’s emotional experience of their life, including their positive and negative emotions. For a long time, scholars have paid more attention to negative emotions, such as depression and anxiety, in children and adolescents, but less attention to positive emotions such as happiness, joy, satisfaction, and optimism.

Recently, with the increasing amount of research in positive psychology, SWB has received extra attention (Diener et al., 2018). A large body of literature mainly regards SWB as an outcome and explores the factors that impact it. However, an interesting topic that has been studied recently involves not only the causes of SWB but also its consequences (Diener et al., 2017). Do people behave differently when they are more or less happy? The general answer is that active SWB appears to be beneficial for numerous life activities, such as social relationships, prosocial behaviors, and PEBs (Diener et al., 2018).

In the realm of PEBs, people high in SWB outperform others (Diener et al., 2018). They are more likely to sacrifice some of their interests, such as give more of their time or money, to meet the need for environmental protection (Kushlev et al., 2021). Sulemana (2016) used 18 countries’ data to verify that the happier people are, the more willing they are to make income sacrifices to protect the environment. Consistent with the results of Wang and Kang (2019), people’s life satisfaction can indeed motivate them to participate in PEBs. PEBs commonly include environmental protection behaviors in the private sector, such as green consumption, energy-saving housing, etc., and those in the public domain, such as actively participating in environmental protection organizations, environmental parades, petitions, etc. Ouyang et al. (2022) further confirmed that SWB has a positive impact on both private PEBs and public PEBs among residents of rural China. Nguyen et al. (2022) divided SWB into psychological wellbeing and social wellbeing, and used data from Vietnam to prove that two different types of wellbeing both can promote individuals’ pro-environmental consumption behaviors. Nevertheless, the available literature is focused on adults and research on the adolescents has yet to be explored.

The existing literature suggests that the relationship between PEBs and age is inverted U-shaped (Krettenauer, 2017). In childhood and adulthood, there is a better performance in terms of PEBs with increasing age (Otto and Kaiser, 2014; Krettenauer, 2017). However, in adolescence, there is a decline in PEBs with age (Negev et al., 2008; Olsson and Gericke, 2016; Wray-Lake et al., 2016; Krettenauer, 2017; Krettenauer et al., 2020). This phenomenon is known as “time out” (Kahn and Kellert, 2002) or “adolescent dip” (Olsson and Gericke, 2016). Adolescence being a stage of dramatic development, adolescents are eager to be free from authority and the value of social responsibility decreases at this age (Wray-Lake et al., 2016). Negev et al. (2008) found that sixth graders in Israel exhibited significantly higher PEBs than 12th graders. This result is also confirmed among Swedish ninth and twelfth grades (Olsson and Gericke, 2016). That is, PEBs decline in adolescence. Actually, Wray-Lake et al. (2016) suggested there was a decline in socially responsible values throughout adolescence. Their comparative study of elementary, middle, and high school students in the United States suggested that older adolescents might be less inclined to daily environmental protection (Wray-Lake et al., 2017). This result is also consistent with the findings of Finnish ninth graders that adolescents are not very interested in big environmental issues (Uitto and Saloranta, 2010; Uitto et al., 2011).

The causes of adolescent decline are not fully understood. Nonetheless, some studies suggest brain development and associated psychological changes as potential contributory factors (Olsson and Gericke, 2016). As a transitional stage of growth and development between childhood and adulthood, adolescence is also a developmental period of increased moral sensitivity (Spear, 2000; Steinberg, 2005; Krettenauer, 2017). During this stage, the adolescent brain continues to change and mature, but there are still many differences compared to adults. This is largely because the frontal lobes are the last areas of the brain to mature, and they do not complete development until a person is in their 20 s (Romine and Reynolds, 2005). The frontal lobe plays an important role in coordinating complex decision-making, impulse control and the ability to consider multiple options and consequences (Fuster, 2002). Adolescents might lack the ability to apply it. When impulses come into play, strong emotions often continue to drive their decisions (Casey et al., 2008). Therefore, emotion is possible an important factor influencing their PEBs.

So far, it is not clear whether the SWB of adolescents can promote their PEBs, and this puzzle needs to be tested empirically. According to Brown and Kasser (2005), their data on adolescents aged 10−18 from the U.S. indicated individuals higher in SWB reported more ecologically responsible behaviors. However, there are a few caveats to supposing that SWB is beneficial for adolescents’ PEBs. We still know little about the cross-cultural transferability of the findings (Diener et al., 2018). Most studies have been conducted in highly economically developed western countries, for instance, the USA, Canada, Sweden, Finland, German, etc., (Negev et al., 2008; Uitto and Saloranta, 2010; Uitto et al., 2011; Liefländer and Bogner, 2014; Olsson and Gericke, 2016; Wray-Lake et al., 2016; Wright et al., 2021). Since SWB is highly valued in these countries, we do not know whether the findings also apply to the developing countries (Sulemana, 2016). In this paper, we use an international dataset from PISA 2018 to test whether 15-year-old students’ SWB significantly impacts their PEBs. Considering the above, we propose the following hypothesis:

*H1. SWB can positively improve PEBs in adolescents.*

### The mediating role of self-efficacy in the relationship between SWB and PEBs

When seeking a better understanding of whether SWB leads to PEBs and the elements that spur such behaviors, there is value in turning to the mechanisms by which SWB influences PEBs.

A great deal of research has been conducted by scholars around the relationship between SWB and PEBs. Previous studies have focused on whether the correlation between the two is significant (Kushlev et al., 2020, 2021; Nguyen et al., 2022). There is also some literature that further explores how SWB affect PEBs and what the mechanisms of action between the two are (Wang and Kang, 2019; Ouyang et al., 2022). Wang and Kang (2019) proposed that environmental concerns play a mediating role between SWBs and PEBs. Those with a high SWB care more about the environment and are therefore more willing to take practical action to protect it. Ouyang et al. (2022) study of Chinese rural residents suggested that the unique “acquaintance society” in rural areas emphasized the role of networks and social links, and that people with higher SWB tend to have a higher sense of social responsibility and altruism, thus enhancing their PEBs. Nevertheless, adolescence is an intense and often stressful period of development (Arnett, 1999; Pervanidou and Chrousos, 2012). Adolescents are not too concerned about environmental issues (Kahn and Kellert, 2002; Negev et al., 2008; Uitto et al., 2011; Otto and Kaiser, 2014; Abraham et al., 2015; Wray-Lake et al., 2017). They are at a particular stage of social responsibility “moratorium” (Erikson, 1994), and it is clear that existing research has not paid enough attention to them.

Adolescence is a necessary stage for adolescents to separate emotionally from their parents and establish independent self-values. At this stage, adolescents try to escape from authority and have a reduced sense of social responsibility. However, they are more concerned with self-efficacy. As a motivation concept, self-efficacy refers to beliefs about one’s contentment with unusual situations (Nicholls et al., 1989; Pajares, 1997; Schunk and Pajares, 2002; Briones and Tabernero, 2012). People with higher self-efficacy generally have higher resilience and persistence in following through (Saribas et al., 2014). Corresponding to the self-determination theory (SDT) of Ryan and Deci (2000), greater intrinsic motivation induces a range of adaptive advantages (Ryan et al., 1997), including enhanced behavioral effectiveness and greater wellbeing (Ryan and Deci, 2000). This is consistent with the findings of Briones et al. (2005), who revealed a direct relationship between SWB and self-efficacy in adolescents. Yuan (2015) found an indirect relationship between psychological wellbeing and self-efficacy through the mediated pathway of inspiration.

According to the norm activation model (NAM) of Schwartz and Howard (1981), individuals’ assessments of anticipatory impairments influence the evolution of their behavioral outcomes. These anticipatory barriers arise from assessments of self-efficacy and perceptions of anticipatory self-efficacy. Value-belief-norm (VBN) theory, put forward by Stern et al. (1999), incorporates value theory based on the mediation model of NAM, emphasizing the influence of self-efficacy perception on behavioral consequences. In the theory of planned behavior (TPB), perceived behavioral control (PBC) is supposed to be based on sensible control beliefs, which can moderate the influence of attitude and subjective norms on intention (Ajzen, 2020). Self-efficacy, as a form of PBC, refers to the degree to which an individual believes that his or her behavior is under control (Ajzen, 2002).

Numerous studies have shown that self-efficacy can be a direct or indirect predictor of PEBs (De Young, 1985; Tabernero and Hernández, 2010; Saribas et al., 2014; Abraham et al., 2015; Sawitri et al., 2015; Kaida and Kaida, 2016a,b, 2019; Lee and Tanusia, 2016). For instance, Kaida and Kaida (2016b) found that self-efficacy was related to PEBs. De Young (2000) elaborated that the human inclination toward competence helped to promote environmentally responsible behavior. Meinhold and Malkus (2005) further explored self-efficacy’s mediating effect on the environmental attitude-behavior relationship, finding that the relationship between pro-environmental attitudes and behaviors was stronger among adolescents with high levels of self-efficacy. Based on the above arguments and evidence, we expect SWB to contribute to enhanced self-efficacy, with this intrinsic satisfaction in turn being translated into actual environmental actions. Thus, we put forward the following hypothesis:

*H2. Self-efficacy has a positive effect on adolescents’ SWB, and self-efficacy has a mediating role in the impact of adolescents’ SWB on their PEBs.*

### Moderators: Peer relationships and multicultural values

On the one hand, peer relationships are one of the most significant features of adolescence (Brown, 2004; Brown and Larson, 2009). As adolescence approaches, the extra struggle for autonomy and time spent with peers increases and time spent with family decreases (Allen and Loeb, 2015). When adolescents grapple with increasingly abstract and complex social issues, they often seek a stable peer group as support for emotional management (Savin-Williams and Berndt, 1990; Roseth et al., 2008; Brown and Larson, 2009). Positive peer relationships stem from the recognition of equality and the tendency to provide emotional support. Positive and supportive peer relationships can promote healthy emotional development and mental health in adolescence (Brown, 2004; Roseth et al., 2008).

Social interactions with peers have been linked to a range of prosocial actions (Wentzel, 2014). Silva and Rodríguez (2022) suggested a significant correlation between pro-social behavior and PEBs. Adolescents who have a stable sense of identification with their peers tend to be more understanding and altruistic (Kerpelman and Pittman, 2001; Portt et al., 2020). Accordingly, we put forward the following hypothesis:

*H3. Peer relationships moderate the positive relationship between SWB on PEBs; such that SWB provides more positive effect to PEBs among adolescents with better peer relationships.*

On the other hand, Multicultural values are at the heart of prosocial behavior (Padilla-Walker et al., 2022). In the context of globalization, as adolescents are growing up more aware of diverse cultural beliefs and behaviors, they increasingly develop the multicultural identities (Arnett Jensen, 2003). Multicultural values help to deepen adolescents’ identification with ethical norms and thus enhance their pro-social behavior (Carlo and Padilla-Walker, 2020). Given that multicultural values influence prosocial behaviors—actions that benefit others (Carlo and Padilla-Walker, 2020), such as how people use natural resources or their willingness to adopt sustainable behaviors (Park et al., 2007)—multicultural values may play a significant role in how adolescents deal with environmental issues. In other words, adolescents with multicultural values are more tolerant of different cultures and may be more likely to develop prosocial behaviors. Therefore, we put forward the following hypothesis:

*H4. Multicultural values moderate the positive relationship between SWB on PEBs; such that SWB provides more positive effect to PEBs among adolescents with stronger multicultural values.*

## Methodology

### Data

Our primary data source is the PISA 2018. PISA, a triennial assessment that launched in 1997, is a comprehensive assessment of 15-year-old school students in member countries and their partner countries or economies by the Organisation for Economic Co-operation and Development (OECD). Which assesses the extent to which 15-year-old students have acquired the knowledge and skills essential for full participation in modern societies (OECD, 2019).

PISA 2018 is selected because it is the widest international survey of adolescents, which contains the most comprehensive multi-item indices of SWB and PEBs. PISA 2018 can maximize cross-cultural comparability by choosing clear, translatable, and where possible quantifiable response formats (OECD, 2019). Most international SWB assessments have so far focused on adult populations, with few surveys conducted with adolescents (OECD, 2019). Moreover, many of these surveys focus on specific subgroups rather than on the general adolescent populations (Casas, 2011). PISA 2015 was the first international large-scale assessment of adolescents’ wellbeing included a few questions on their SWB. However, the set of questions were limited in scope (OECD, 2017). In PISA 2018, a separate wellbeing questionnaire encompassing questions covering the entire wellbeing construct could be a building block for international benchmarks on adolescent wellbeing. Additionally, multi-indices, rather than single-item, were used to measure adolescents’ SWBs and PEBs in PISA 2018. The multi-item measurement approach is helped to increase the robust across nations and economies (Casas et al., 2012).

In PISA 2018, SWB and PEBs-related questions were surveyed in only eight economies: Ireland, Spain, Bulgaria and Serbia in Europe, the United Arab Emirates and Hong Kong in Asia, and Mexico and Panama in the Americas. These countries or economies are located on different continents, in both developed and developing countries or regions. Excluding invalid data, we obtain 56,374 valid samples which form the dataset for this paper.

### Dependent variable: PEBs

The main goal of this study is to understand how adolescents’ SWB affects their PEBs. To address this issue, we must clarify the indicators used to measure PEBs. In the questionnaire administered to students for PISA 2018, there are five questions regarding PEBs (see Table 4). The respondent can answer 1 or 2 to each of these questions, where “1” indicates yes, and “2” no. We adjust the assignment, assigning a value of 0 to no and a value of 1 to yes. Then, we compute the total score for the five items. The variable PEBs thus takes scores from 0 to 5.

### Core explanatory variable: SWB

In PISA 2018, there are five questions used to measure SWB (see Table 2). The responses can range from 1 to 4, where 1 refers to “never,” 2 to “rarely,” 3 to “sometimes,” and 4 to “always.” The method of measurement is a four-point Likert-type. Thus, we test the reliability and validity of the scale (see Table 2). Cronbach’s α is 0.836 (> 0.70), which implies well internal consistency. The KMO value and Bartlett’s test of sphericity show satisfactory results (KMO = 0.856; χ^2^ = 113,627.9, *p* < 0.001), which indicate the suitability of factor analysis. Factor loadings (FL) of all items are higher than 0.5 indicate good construct validity. The composition reliability (CR) value is 0.899 (> 0.60) and the average variance extracted (AVE) is 0.619 (> 0.50), demonstrating the convergent validity is acceptable (Hair et al., 2018).

**TABLE 2:** Reliability and validity of the measurement scale of SWB.

Variable	Measure items	FL	Cronbach’s α	CR	AVE
SWB	How often do you feel Happy?	0.834	0.836	0.889	0.619
	How often do you feel Lively?	0.761			
	How often do you feel Proud?	0.622			
	How often do you feel Joyful?	0.844			
	How often do you feel Cheerful?	0.848			

### Mediating variable: Self-efficacy

The self-efficacy variable is formulated from the students’ answers to five questions (see Table 3). Answers can range from 1 to 4, where 1 means “strongly disagree,” 2 means “disagree,” 3 means “agree,” and 4 means “strongly agree.” Cronbach’s α is 0.792 (> 0.70), which suggests the reliability of internal consistency. The KMO value and Bartlett’s test of sphericity show satisfactory results (KMO = 0.811; χ^2^ = 75,661.0, *p* < 0.001), which indicate the suitability of factor analysis. FL of all items are higher than 0.5, CR > 0.8, and AVE > 0.5. The convergent validity is acceptable (Hair et al., 2018).

**TABLE 3:** Reliability and validity of the measurement scale of Self-efficacy.

Variable	Measure items	FL	Cronbach’s α	CR	AVE
Self-efficacy	I usually manage one way or another.	0.687	0.792	0.858	0.548
	I feel proud that I have accomplished things.	0.740			
	I feel that I can handle many things at a time.	0.735			
	My belief in myself gets me through hard times.	0.762			
	When I’m in a difficult situation, I can usually find my way out of it.	0.774			

**TABLE 4:** Descriptive statistics.

Variable	Measures	N	SD	Mean	Min	Max
PEBs	I reduce the energy I use at home to protect the environment.	56,374	0.400	0.800	0	1
	I choose certain products for ethical or environmental reasons, even if they are a bit more expensive.	56,374	0.500	0.514	0	1
	I boycott products or companies for political, ethical, or environmental reasons.	56,374	0.464	0.315	0	1
	I sign environmental or social petitions online.	56,374	0.471	0.333	0	1
	I participate in activities in favor of environmental protection.	56,374	0.500	0.488	0	1
SWB	How often do you feel Happy?	56,374	0.652	3.353	1	4
	How often do you feel Lively?	56,374	0.727	3.216	1	4
	How often do you feel Proud?	56,374	0.809	2.977	1	4
	How often do you feel Joyful?	56,374	0.717	3.302	1	4
	How often do you feel Cheerful?	56,374	0.701	3.339	1	4
Self-efficacy	I usually manage one way or another.	56,374	0.686	3.043	1	4
	I feel proud that I have accomplished things.	56,374	0.684	3.268	1	4
	I feel that I can handle many things at a time.	56,374	0.728	2.988	1	4
	My belief in myself gets me through hard times.	56,374	0.809	2.980	1	4
	When I’m in a difficult situation, I can usually find my way out of it.	56,374	0.706	3.073	1	4
PE	This school year, on average, on how many days do you attend physical education classes each week?	56,374	3.079	1.194	1	8
Gender	Male = 0; Female = 1	56,374	0.500	0.508	0	1
Grade	Student International Grade	56,374	0.664	9.727	7	12
Health	How is your health? (Poor = 1; Fair = 2; Good = 3; Excellent = 4)	56,374	0.693	3.261	1	4
Environmental knowledge	Explain how carbon-dioxide emissions affect global climate change. (I couldn’t do this = 1; I would struggle to do this on my own = 2; I could do this with a bit of effort = 3; I could do this easily = 4).	56,374	0.952	2.758	1	4
Mother’s education	She did not complete < ISCED level 1> = 1; completed < ISCED level 1 > = 2; < ISCED level 2 > = 3; < ISCED level 3B, 3C > = 4; < ISCED level 3A > = 5; < ISCED level 4 > = 6; < ISCED level 5B > = 7;< ISCED level 5A > = 8; < ISCED level 6 > = 9	56,374	2.231	6.130	1	9
Father’s education	Same with mother’s education.	56,374	2.295	6.069	1	9
Region	Which of the following definitions best describes the community in which your school is located? [A village, hamlet or rural area (fewer than 3,000 people) = 1; a small town (3,000 to about 15,000 people) = 2; a town (15,000 to about 100,000 people) = 3; a city (100,000 to about 1,000,000 people) = 4; a large city (with over 1,000,000 people) = 5]	56,374	1.136	3.315	1	5
GDP per capita	GDP per capita of the respondent’s country, in 2018, obtained from the International Monetary Fund	56,374	16,338	32,634	7,252	78,989
OECD	Non-OECD members = 0; OECD members = 1	56,374	0.499	0.532	0	1
Peer relationships	How many days a week do you usually spend time with your friends right after school? (0 days = 1; 1 days = 2; 2 days = 3; 3 days = 4; 4 days = 5; 5 days = 6; 6 days = 7)	56,374	1.764	3.766	1	7
Multicultural values	How well does the following describe you: I respect the values of people from different cultures? (Not at all like me = 1; Not much like me = 2; Somewhat like me = 3; Mostly like me = 4; Very much like me = 5)	56,374	0.948	4.342	1	5

### IV variable

The days of PE per week at school is IV variable of this paper. In PISA 2018, there is a question about PE: “This school year, on average, on how many days do you attend physical education classes each week?” The respondent can answer 1 to 8, representing 0 to 7 days, respectively, where “1” represents 0 days, and “8” represents 7 days.

### Control variables

Prior studies have shown that the PEBs of adolescents are influenced not only by individual demographic characteristics, but also by their family, school, and institutional context. Accordingly, we include control variables from different dimensions. The first is the individual dimension, including demographic characteristics (e.g., gender, grade, health), and environmental cognition (e.g., environmental knowledge). The second is the family dimension, for which we control the educational level of the parents. The third is the school dimension. We control the influence of the population size of the region where the school is located. Finally, we consider national-level effects, namely the 2018 per capita GDP of the economy from which the respondents come. In addition, considering that PISA is organized by the OECD, the economies of the respondents are either OECD members or not. OECD members are usually considered developed countries, while non-OECD members are considered developing countries (Gozgor et al., 2020; Myovella et al., 2020). Therefore, we use a dummy variable indicating this. Table 4 provides the measures and descriptive statistics of the variables used in this paper.

### Model specification

The dependent variable, PEBs, is an ordinal variable. Thus, as recommended by Wang and Kang (2019), and Ouyang et al. (2022), we employ an ordered probit model to examine the influence of adolescents’ SWB on their PEBs. Furthermore, considering that PISA 2018 is an international survey carried out in different countries or economies around the world, we use the country or economy as a fixed effect to control the possible impact of the economy’s institutional context on adolescents’ PEBs. The model is as follows:


(1) $$PEB_{ie}={\text{β}}_{0}+{\text{β}}_{1}SWB{\;}_{ie}+{\text{β}}_{2}X_{ie}+{\text{λ}}_{i}+{\text{ε}}_{ie}$$

where *i* and *e* represent the student and his or her country/economy, respectively. *PEB*_*ie*_, the dependent variable, stands for the PEBs of student *i* residing in country/economy *e*. *SWB _*ie*_*, the independent variable, represents the SWB of student *i* residing in country/economy *e*. *X*_*ie*_ is the set of control variables. Finally, *λ_*i*_* is the country/economy-fixed effect and *ε_*ie*_* is an error term.

## Results

### General results

Table 5 reports the regression results for the impact of adolescents’ SWB on their PEBs. As shown, adolescents’ SWB has a positive and significant impact on their PEBs (β = 0.193, *p* < 0.01). Moreover, control variables are added in column (2), and the influence of SWB on PEBs in adolescents remains significant and positive (β = 0.198, *p* < 0.01). Thus, H1 is verified.

**TABLE 5:** Impact of adolescents’ SWB on their PEBs.

	PEBs (1)		PEBs (2)	
	**Coef.**	***Z*** **-value**	**Coef.**	***Z*** **-value**
SWB	0.193^***^	22.15	0.198***	21.27
Gender			−0.053***	−5.93
Grade			0.031***	4.10
Health			0.032***	4.44
Environmental knowledge			0.099***	20.14
Mother’s education			0.009***	3.74
Father’s education			0.011***	4.43
Region			−0.057***	−14.24
GDP per capita			0.000***	5.08
OECD			−0.520***	−54.78
Fixed effect	YES		YES	
Observations	56,374		56,374	
*R* ^2^	0.004		0.0268	

***p < 0.01.

As to the internal individual demographics, it is clear that participation in PEBs differs substant0ially between girls and boys. Among adolescents, girls with high SWB perform worse in PEB than boys (β = −0.053, *p* < 0.01). Compared to girls, boys may be more willing to be close to nature and prefer more outdoor exploration activities. The respondents are all 15-year-olds but come from different grades, the lowest being grade 7 and the highest being grade 12. Our results show that the higher adolescents’ grades, the higher their willingness to protect the environment (β = 0.031, *p* < 0.01). Correspondingly, adolescents with more environmental knowledge exhibit stronger environmental protection actions (β = 0.099, *p* < 0.01). This shows that, the higher the grade the student attains, the more environmental knowledge they may acquire, the deeper their understanding of the importance of environmental protection will be, and the more willing they will be to take actions to support environmental protection. Additionally, consistent with the expectations of the existing literature, healthier adolescents are more actively involved in environmental protection (β = 0.032, *p* < 0.01).

As to the external family and social institutional context, first of all, both the education level of the father (β = 0.011, *p* < 0.01) and that of the mother (β = 0.009, *p* < 0.01) have a significant positive impact on the PEBs of adolescents. In addition, the population size of the region in which the school is located shows a negative correlation with the students’ PEBs (β = −0.057, *p* < 0.01). Possibly, cities with large population densities tend to have a faster pace of life, greater competitive pressure, and greater academic pressure on students. This may lead to students’ attention being occupied by learning, while activities such as protecting the environment, which may be seen as hindering their studies, are neglected.

According to the affluence hypothesis, richer societies have higher environmental concerns (Inglehart, 1995). Our results confirm that the higher the GDP per capita in the economy where adolescents live, the more willing they will be to protect the environment (β = 0.000, *p* < 0.01). Last but not least, our results show that there is a negative correlation between SWB and PEBs among students from OECD countries or economies (β = −0.052, *p* < 0.01). The OECD plays a key role in promoting the formulation and implementation of sustainable development policies in members which are almost the developed countries. Citizens of these countries may be more satisfied with the environmental policies and their effects, which could reduce their likelihood of participating in PEBs. Meanwhile, environmental problems in non-OECD countries or economies may be more prominent. When people are dissatisfied with government policies, they are more likely to self-manage and actively participate in environmental protection activities. This may be a case of “national advancement and private retreat” or “national retreat and private advancement.”

### Robustness checks

To verify the stability and reliability of the general regression results, we run robustness tests. From the earlier review, it can be seen that a potential predicament when studying SWB is that of causality. That is, is happiness the result of PEBs in adolescents or an antecedent of PEBs? Likewise, although we consider as many control variables as possible, that may affect PEBs in adolescents, we cannot completely avoid the possibility of omitted variables. Due to possible measurement errors, the parameter estimates obtained by the simple ordered Probit model may be biased and inconsistent. As noted by Sulemana (2016), Wang and Kang (2019) and Ouyang et al. (2022), we need to find an ideal IV to control the endogeneity.

In the field of education, physical education is a discipline that contributes to facilitating the wellbeing and health of adolescents (Bailey et al., 2009; Luna et al., 2019). A large number of existing studies have shown that physical activity plays a unique role in improving human health and SWB (Gauvin, 1989; Stathi et al., 2002; Martin Ginis et al., 2010; Pawlowski et al., 2011; Wicker and Frick, 2015; Wiese et al., 2018; Buecker et al., 2021; McCurdy et al., 2022; Zhang et al., 2022). Among adolescents, this conclusion is supported by numerous studies (Valois et al., 2004; Brooks and Magnusson, 2007; Smith et al., 2018; Luna et al., 2019; Wright et al., 2021). Brooks and Magnusson (2007) revealed that participation in physical activities by adolescent girls in the UK significantly improved their health and SWB. The results of Luna et al. (2019) evaluation of the pilot reform of the physical education curriculum for 12−15-year-old youth in Spain showed that the optimized physical education curriculum design significantly impacted the SWB of adolescents. Results from Wright et al. (2021) empirical study of 13−19-year-olds in the UK suggested that physical activity could counteract the threat of fear to adolescent health and SWB during the COVID-19 Pandemic.

Given that adolescents spend the majority of their time in school, PE could have a positive impact on students’ social and emotional competencies (Luna et al., 2019). Therefore, this paper selects the days of PE per week at school as the IV. For students, PE is usually a rare opportunity to have fun and relax at school, and more PE will undoubtedly increase their SWB. Meanwhile, the setting of PE is arranged by the school or possibly even the local education authority, and is thus an exogenous variable that cannot be controlled by students.

Drawing on Sulemana (2016), Wang and Kang (2019), we use the IV Probit model and estimate it by the 2SLS method to obtain an accurate estimation of the contribution of adolescents’ SWB to their PEBs. Table 6 reveals that the *F*-value in the first-stage regression is greater than the suggest empirical value of 10, ruling out the possibility that PE is a weak IV. After adding the IV, adolescents’ SWB still has a positive impact on their PEBs (β = 2.705, *p* < 0.01). Thus, the results of this paper are robust.

**TABLE 6:** Two-stage least squares method estimates.

	PEBs (1)		PEBs (2)	
	**1^st^ stage**		**2^nd^ stage**	
	**Coef.**	***T*** **-value**	**Coef.**	***Z*** **-value**
SWB			2.705***	9.48
Physical education classes	0.027***	12.21		
Control variables	YES		YES	
Fixed effect	YES		YES	
Observations	56,374		56,374	
Wald chi2 (11)			2,773.54	
Prob > chi2 (F)	0.000		0.000	
*F*-value	843.37			

*** *p* < 0.01.

### Mechanism analysis: Mediating role of self-efficacy

To better understand the mechanism of influence of adolescents’ SWB on their PEBs, we further explore the pathway by which SWB affects PEBs in adolescents. Table 7 presents the mediating effect of self-efficacy in the relationship between the SWB and PEBs of adolescents. Consistent with Briones et al. (2005), Yuan (2015), we find that SWB has a significant positive effect on adolescents’ self-efficacy [column (2)]. That is, adolescents who report being happier generally have higher levels of self-efficacy. After adding the mediating variable, the results show that self-efficacy is beneficial for the PEBs of adolescents, and their SWB still has a significant positive effect on their PEBs [(column (3)]. Specifically, self-efficacy can be regarded as an important path through which adolescents’ happiness can play a critical role in promoting their PEBs. Therefore, H2 is verified.

**TABLE 7:** Mediating role of self-efficacy.

	PEBs (1)		Self-efficacy (2)		PEBs (3)	
	**Coef.**	***Z*** **-value**	**Coef.**	***Z*** **-value**	**Coef.**	***Z*** **-value**
SWB	0.198***	21.27	0.796***	75.47	0.171***	17.00
Self-efficacy					0.076***	7.43
Control variables	YES		YES		YES	
Fixed effect	YES		YES		YES	
Observations	56,374		56,374		56,374	
*R* ^2^	0.027		0.062		0.027	

*** *p* < 0.01.

### Moderating effect: Peer relationships and multicultural values

We use the question from PISA 2018, “How many days a week do you usually spend time with your friends right after school?,” as an observed variable reflecting peer relationships among adolescents (the descriptive statistics see Table 4). Specifically, we allocate the respondents who spend 4 to 6 days per week with their classmates after school to the “better peer relationships” group (*Peer relationships* = 1), and those who spend 0 to 3 days with them to the “general peer relationships” group (*Peer relationships* = 0). We employ the interaction item to test the moderation effect. Column (1) in Table 8 shows that the coefficient of SWB*Peer relationships (β = 0.010) is significant at the 1% level, implying that adolescents with better peer relationships have positive moderation effect on the relationship between SWB on PEBs. The result is consistent with Wang et al. (2021). Thus, H3 is verified.

**TABLE 8:** Moderating effect of peer relationships and multicultural values.

	Peer relationships (1)		Multicultural values (2)	
	**Coef.**	***Z*** **-value**	**Coef.**	***Z*** **-value**
SWB	0.150***	13.66	0.149***	8.39
SWB*Peer relationships	0.010***	7.54		
Peer relationships	0.009	0.55		
SWB*Multicultural values			0.008**	2.45
Multicultural values Control variables			0.193***	10.74
	YES		YES	
Fixed effect	YES		YES	
Observations	56,374		56,374	
*R* ^2^	0.028		0.030	

* *p* < 0.1, ** *p* < 0.05, *** *p* < 0.01.

According to the question in PISA 2018, “How well does the following describe you: I respect the values of people from different cultures?,” we divide the respondents into the group of general multicultural values and stronger multicultural values. In this question, the respondents can answer 1 to 5, where 1 refers to “Very much like me,” 2 to “Mostly like me,” 3 to “Somewhat like me,” 4 to “Not much like me,” 5 to “Not at all like me.” As this answer is set to reverse the assignment, we readjust the assignment. That is, 1 means “Not at all like me” while 5 means “Very much like me” (the descriptive statistics see Table 4). We then classify the respondents who select “Very much like me” into the stronger group (*Multicultural values* = 1) and the remainder into the general group (*Multicultural values* = 0). The interaction item is used to test the moderation effect. In column (2) of Table 8, we find that the coefficient of SWB*Multicultural values (β = 0.008) is significant at the 1% level, indicating that adolescents with stronger multicultural values have positive moderation effect on the relationship between SWB on PEBs (see Table 8). The result is consistent with Park et al. (2007). Therefore, H4 is verified.

## Discussion

In this paper, we focus on whether adolescents’ positive affect, SWB, contributes to their PEBs. Our findings present evidence of the effects of adolescents’ SWB on their PEBs. We employ international data from PISA 2018, administered by the OECD, and implement an IV approach to alleviate concerns about the endogeneity of SWB and its possible association with unobserved factors affecting adolescents’ PEBs. Our instrumental strategy uses a non-individually-controlled exogenous variable, the days per week that the school offers PE. The IV Probit model results show that the improvement of adolescents’ SWB significantly increases their participation in PEBs. Additionally, our findings indicate that adolescents’ self-efficacy is the potential pathway by which this happens. That is, self-efficacy plays a partial mediating role in the effect of SWB on PEBs in adolescents. Finally, we further analyze the moderating effects of adolescents’ peer relationships and multicultural values. The results show that adolescents with better peer relationships and stronger multicultural values have a greater positive coefficient for the relationship between their SWB and their PEBs.

### Theoretical contributions

Overall, this paper presents a comprehensive theoretical framework for the PEBs from the perspective of positive psychology. Firstly, our findings enrich theoretical research in environmental psychology. Several scholars have confirmed the causal relationship between PEBs for positive emotions in adult populations, and this paper builds on these studies to confirm that this causal relationship still holds in adolescent populations using data from eight countries or economies. Given that existing theoretical frameworks in environmental psychology ignore the value of emotions, our study provides empirical evidence for the role of positive emotions in PEBs.

Additionally, this paper further enriches the study of adolescent PEBs from a positive psychology perspective. Most existing studies focus on objective factors at the individual or social level, while subjective factors are scarcely explored (Barbaro and Pickett, 2016; Molinario et al., 2020; Scopelliti et al., 2022). To the best of our knowledge, only Solano-Pinto et al. (2020) have empirically tested whether there is a reciprocal influence between children’s wellbeing and connectivity with nature which may promote their PEBs. Our findings validate the causal relationship of SWB on PEBs and move research in this area forward.

Last but not least, this paper provides new evidence for the reverse causality of SWB to PEBs. In previous studies, some scholars have viewed SWB as outcomes and some as predictors. In order to address the endogeneity of SWB, the search for the ideal IV is the big question that positive psychology is eager to address. The use of PE classes in schools as an IV in this paper satisfies both the exogeneity and relevance of the IV. On the one hand, PE is exogenous. It is set by the school or even the government and is not influenced by the individual. On the other hand, PE has a significant effect on PEBs in adolescents. Thus, our findings provide new ideas for the study of adolescent psychology.

### Managerial implications

Our findings should also be taken into consideration when discussing encouraging youth groups to participate in environmental protection. This paper provides new insights into addressing the decline in environmental awareness among adolescents. The promotion of SWB in adolescents not only helps to promote PEBs, but also benefits the development of adolescents. First of all, Governments should strengthen the monitoring and evaluation of students’ SWB, and guide schools, families, and society to pay more attention to the positive emotions of adolescents. As indicated by the findings of this paper, SWB is a significant element to consider in policy discussions around PEBs. Adolescents who are satisfied with their lives are more likely to participate in PEBs. Thus, policymakers should consider policies that can directly enhance the levels of SWB among adolescents.

Furthermore, Policymakers, educators and families should create more space and opportunities for young people to grow. Adolescence, as a special time of transition to adulthood, is a time when young people are more focused on their own experiences and emotions. Our study also confirms that self-efficacy plays an important role between adolescent SWBs and PEBs. Besides, they are eager to be free from bondage and to confront authority. The impact of peer relationships on them is increasingly evident. Having better peer relationships not only contributes to their health but also to their enjoyment of participating in social activities. Policymakers should encourage schools and families to create more opportunities for adolescents to spend leisure time with their peers.

Eventually, the government ought to develop programs to strengthen the cultivation of multicultural values in adolescents, which is conducive to cultivating more open and tolerant teenagers, thereby helping to promote their prosocial behaviors and encourage them to maintain the safety of the environment.

### Limitations and future research

This study is not without limitations. Firstly, the data used to measure PEBs is self-reported, which means it is potentially subject to measurement error. Therefore, future research should focus on multidimensional measures of PEBs. Furthermore, due to data limitations, a global general study is clearly beyond the scope of this paper. This is because, PISA 2018 was only conducted in eight countries or economies with questionnaires related to SWBs and PEBs. Therefore, future studies will require data from additional countries or economies to verify the applicability and generality of the findings. In particular, comparative studies across countries should be conducted to examine the impact of the national level on the relationship between adolescents’ SWB and PEBs.

Finally, the survey objects of these data are 15-year-old school students in the year of the survey’s implementation, and there is a lack of longitudinal data from long-term follow-up surveys. There might be non-linear effects of adolescents’ SWB on their PEBs, and there could be unobserved heterogeneity. Thus, future research would benefit from longitudinal experiments with long-term follow-up investigations. Moreover, finding ideal IVs can help to address endogeneity issues and thus enhance the stability of causality estimates. Given the strong subjective nature of SWB, external objective factors that are not influenced by individuals can be used for IV, such as external policy shocks, natural environmental factors, historical events, etc. It is also important to exclude any possible correlation between these external factors and PEBs. Future research may explore more ideal IVs to solve endogenous problems.

## Conclusion

How to combat the “adolescent dip” in PEBs is a great challenge for policymakers and educators. The present work suggests that adolescents’ SWB is positively associated with PEBs. That is, adolescents who have higher SWB perform better in terms of PEBs. Moreover, self-efficacy partially mediates the relationship between SWBs and PEBs in adolescents. Furthermore, the effect of SWB on PEBs is more pronounced for adolescents with better peer relationships and stronger multicultural values. Our findings not only enrich the theoretical framework of environmental psychology but also provide new ideas to address the decline in environmental behaviors during adolescence.

## Data availability statement

Publicly available datasets were analyzed in this study. This data can be found here: The OECD’s Programme for International Student Assessment 2018 at https://www.oecd.org/pisa/data/2018database/.

## Author contributions

MZ: conceptualization, formal analysis, writing — original draft, writing — review and editing, visualization, funding acquisition, and project administration. WZ: formal analysis, data curation, and visualization. YS: conceptualization, formal analysis, writing — original draft, writing — review and editing, funding acquisition, and supervision. All authors contributed to the article and approved the submitted version.
